# Total parathyroidectomy versus total parathyroidectomy with autotransplantation for secondary hyperparathyroidism: systematic review and meta-analysis

**DOI:** 10.1080/0886022X.2017.1363779

**Published:** 2017-08-30

**Authors:** Changjia Li, Liang Lv, Hongqiao Wang, Xufu Wang, Bangxu Yu, Yan Xu, Xiaobin Zhou, Yanbing Zhou

**Affiliations:** a Department of General Surgery, Affiliated Hospital of Qingdao University, Qingdao, China;; b Department of Ultrasound, Affiliated Hospital of Qingdao University, Qingdao, China;; c Department of Nuclear Medicine, Affiliated Hospital of Qingdao University, Qingdao, China;; d Department of Intensive Care Unit, Affiliated Hospital of Qingdao University, Qingdao, China;; e Department of Nephrology, Affiliated Hospital of Qingdao University, Qingdao, China;; f Department of Epidemiology and Health Statistics, Qingdao University Medical College, Qingdao, China

**Keywords:** Secondary hyperparathyroidism, parathyroidectomy, chronic kidney disease, meta-analysis

## Abstract

**Background:** Total parathyroidectomy (tPTX) and total parathyroidectomy with autotransplantation (tPTX + AT) are effective and inexpensive treatments for secondary hyperparathyroidism (sHPT), but we do not know which one is the optimal approach. Therefore, we undertook a meta**-**analysis to compare the safety and efficacy of these two surgical procedures.

**Methodology:** Studies published in English on PubMed, Embase and the Cochrane Library from inception to 27 September 2016 were searched systematically. Eligible studies comparing tPTX with tPTX + AT for sHPT were included and Review Manager v5.3 was used.

**Results:** Eleven studies were included in this meta**-**analysis. Ten cohort studies and one randomized controlled trial (RCT) involving 1108 patients with sHPT were identified. There was no significant difference in the prevalence of surgical complications (relative risk [RR], 1.71; 95% confidence interval [CI], 0.77–3.79; *p* = .19), all-cause mortality (RR, 0.68; 95% CI, 0.33–1.39; *p* = .29), sHPT persistence (RR, 3.81; 95% CI, 0.56–25.95; *p* = .17) or symptomatic improvement (RR, 1.02; 95% CI, 0.91–1.13; *p* = .79). tPTX could reduce the risk of sHPT recurrence (RR, 0.19; 95% CI, 0.09–0.41; *p* < .0001) and reoperation because of recurrence or persistence of sHPT (RR, 0.46; 95% CI 0.24–0.86; *p* = .01) compared with tPTX + AT. Simultaneously, tPTX increased the risk of hypoparathyroidism (RR, 2.63; 95% CI, 1.06–6.51; *p* = .04).

**Conclusions:** We found tPTX and tPTX + AT to be useful methods for sHPT treatment. tPTX was superior for reducing the risk of sHPT recurrence and reoperation than tPTX + AT but, due to a lack of high statistical**-**power RCTs, comparative studies will be needed in the future.

## Background

Chronic kidney disease (CKD) has become a worldwide public**-**health problem [1]. Secondary (renal) hyperparathyroidism (sHPT) is a prevalent complication of CKD [2]. Persistently elevated levels of parathyroid hormone (PTH) and parathyroid hyperplasia are features of sHPT. Hypocalcemia, hyperphosphatemia, and 1,25(OH)_2_D_3_ deficiency play important roles in sHTP [3]. The only way to cure sHPT is kidney transplantation but the number of available kidneys for transplantation is limited. Hence, medical and surgical treatments are the only ways to combat sHPT.

In the early stages, patients can be treated by a low-phosphate diet, phosphate binders, vitamin**-**D analogs, and calcimimetic agents, whereas more advanced stages often require surgical treatment [4]. The calcimimetic agent cinacalcet can achieve identical results to those elicited by surgery. In Germany, the annual cost for cinacalcet (60 mg/day) is ≈€5828.4, whereas the cost of surgery for one person is about €3755.38 [5]. The cost is one disadvantage of cinacalcet. Furthermore, it does not always work. Therefore, surgery is needed in the most advanced stages of sHPT and would be acceptable.

There are three surgical approaches for parathyroidectomy: subtotal parathyroidectomy (sPTX), total parathyroidectomy (tPTX) and total parathyroidectomy with autotransplantation (tPTX + AT). sPTX is resection of 3.5 parathyroid glands, leaving 40**–**80 mg of the most normal**-**appearing parathyroid gland *in situ* [6]. tPTX was first described in 1967, and involves identification and resection of all parathyroid glands. tPTX + AT comprises resection and AT. All parathyroid glands are resected, then the most normal**-**appearing glands are ‘minced’ into 10–201 mm^3^ pieces for AT. The sternocleidomastoid muscle and brachioradialis muscle of the non-dominant forearm as potential sites for AT. Guidelines set by the kidney disease outcomes quality initiative (K/DOQI) [7] and most experts recommend sPTX and tPTX + AT for sHPT, but a consensus on the best operative management is lacking.

K/DOQI [7] guidelines recommend that patients with severe hyperparathyroidism (persistent serum levels of intact PTH >800 pg/mL [88.0 pmol/L]), accompanied with hypercalcemia and/or hyperphosphatemia refractory to medical therapy should receive PTX. However, guidelines set by the Japanese society for dialysis therapy (JSDT) recommend that patients with severe hyperparathyroidism (persistent serum levels of intact PTH >500 pg/mL), accompanied with hypercalcemia (serum calcium >10.0 mg/dL) and/or hyperphosphatemia (serum phosphate >6.0 mg/dL) refractory to medical therapy should receive PTX [8].

Secondary hyperparathyroidism patients usually suffer from bone pain, pruritus, fractures, sleep disorders, restless leg syndrome and ‘shrinking man syndrome’. PTX can reduce the prevalence of all-cause mortality and cardiovascular mortality, and can improve the quality of life (QoL) simultaneously. Jia et al. [9] compared the two procedures up to December 2013, and new articles on this issue have been published since then. The aim of the present study was to compare tPTX with tPTX + AT by carrying out a systematic review and meta**-**analysis.

## Methods

### Data sources and search strategy

Two experienced reviewers designed and carried out the search strategy. We searched PubMed, Embase and Cochrane databases using the following keywords: ‘parathyroidectomy’, ‘hyperparathyroidism, secondary’, ‘hyperparathyroidism, renal’ and ‘kidney disease’. Searching was carried out from the inception of each database to 27 September 2016. In addition, references and cited articles were searched manually to identify other studies meeting the inclusion criteria.

### Inclusion and exclusion criteria

Inclusion criteria were: (i) randomized controlled trials (RCTs), cohorts or case-control studies; (ii) comparison of short-term and long-term (follow-up duration ≥6 months) outcomes between tPTX and tPTX + AT in sHPT; (iii) outcomes included at least one of the following endpoints: all-cause mortality, surgical complications (bleeding and/or voice hoarseness), reoperation prevalence, persistent and/or recurrent hyperparathyroidism, hypoparathyroidism; (iv) results were published in English.

Exclusion criteria were: (i) enrolled patients with primary hyperparathyroidism or tertiary hyperparathyroidism; (ii) enrolled patients underwent repeated surgical procedures; (iii) articles were letters, case reports, reviews, comments, editorials or proceedings. In particular, if one surgical team or institution published more than one article, then repetitive publication was identified. If such duplicated articles could not be distinguished, then the article published most recently or the longest article was selected.

### Data extraction and quality assessment

Two reviewers working independently examined and selected all potentially eligible articles. Disagreements were resolved by discussion with a third reviewer. The following information was extracted in standardized forms: name of the first author; year of publication; location of study; study type; population characteristics (age, sex, number); surgical indications and procedures; duration of follow-up; outcomes. The Newcastle**–**Ottawa scale (NOS) was used to assess the quality of non-randomized studies, whereas the Cochrane assessment tool was used to assess RCTs. The NOS consists of three quality parameters: ‘selection’, ‘comparability’ and ‘outcome’. ‘Quality’ is assessed using a star system: four in the selection domain, two in comparability and three in outcome, making a total of nine stars. A study graded with ≥5 stars is considered ‘high quality’, whereas that with <5 stars is considered ‘low quality’. Two reviewers assessed the quality of each study independently and disagreements were resolved by a third reviewer.

### Statistical analyses

Data were analyzed using Review Manager v5.3.0 (Cochrane Collaboration). For dichotomous scales, data are expressed as relative risk (RR) along with a 95% confidence interval (CI). If there were continuous data of outcomes, then data are presented as the mean difference (MD). We used the *χ*^2^ test and *I*^2^ statistic to assess heterogeneity among studies. *p* < .1 [10] and *I*^2^ > 50% [11] indicated heterogeneity to be significant. Data that were not significantly heterogeneous (*p* > .1, *I*^2^ ≤ 50%) were calculated using a fixed-effects model, whereas heterogeneous data (*p* < .1, *I*^2^ > 50%) were calculated using a random-effects model. Publication bias was assessed using a funnel plot for standard error by effect size (log RR). *p* < .05 was considered significant.

## Results

### Literature search and characteristics

**Figure 1.**
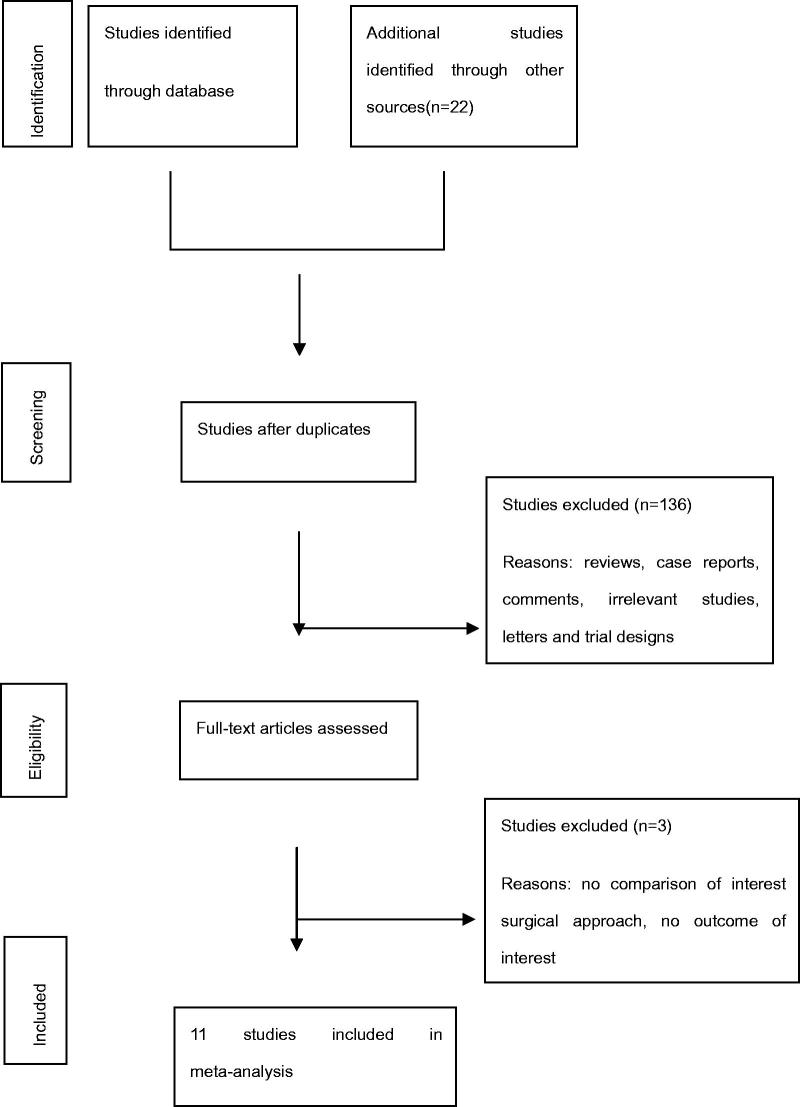
Flow diagram of the identification process for eligible studies.

A flow chart of the selection process is shown in Figure 1. Finally, eleven studies [12–22] were included for studies comparing tPTX with tPTX + AT published between 1991 and 2016. A total of 275 patients were in the tPTX group and 833 patients were in the tPTX + AT group. The baseline characteristics of these included studies and NOS assessments are listed in Tables 1 and 2, respectively. For RCTs, the Cochrane assessment is listed in Table 3.

**Table 1.** Detailed characteristics of studies included in the meta-analysis.

Study	Year	Country	Study design	Study period	Follow up	Sample size	Age (years)	Sex (%, men)	Surgical indication
Higgins et al.	1991	UK	RCS	1997–1990	62 m(4–152) m	tPTX: 9 tPTX + AT: 49 Other: 18	43.2 (26–67)	50	Yes
Nicholson et al.	1996	UK	RCS	1982–1993	24 m	tPTX: 24 tPTX + AT: 13 Other: 11	33–62	50	Yes
Coen et al.	2001	Italy	RCS	NA	3.3 ± 2.3 y(1–10) y	tPTX: 10 tPTX + AT:10 Other: 25	56 ± 11	55.6	No
Ockert et al.	2002	Germany	RCS	1993–1999	23 m (1–49) m	tPTX: 11 tPTX + AT: 11	27–68	45	Yes
Jofré et al.	2003	Argentina	RCS	1990–2002	5.2 ± 3.9 y	tPTX: 19 tPTX + AT: 129	49 ± 14	48.6	Yes
Shih et al.	2009	Taiwan China	RCS	1995–2006	43.1 m	tPTX: 44 tPTX + AT: 14 other:36	50.5 46	48.9	Yes
Conzo et al.	2012	Italy	PCS	1999–2006	1 y	tPTX:20 tPTX + AT:20	52 (26–72)	57.5	Yes
Schneider et al.	2012	Germany	RCS	1976–2009	57.6 ± 2.4 m	tPTX: 32 tPTX + AT: 504 Other: 70	48.5 ± 0.57	53.1	Yes
He et al.	2014	China	RCS	2008–2012	42 m	tPTX: 33 tPTX + AT: 14	46 (28–71)	55.3	Yes
Liang et al.	2015	China	PCS	2010–2014	6 m	tPTX: 21 tPTX + AT: 21 Other: 21	53.2 ± 12.7 (28–72)	60.3	Yes
Schlosser et al.	2016	Germany	RCT	2007–2010	36 m	tPTX: 52 tPTX + AT: 48	49.2 ± 15.6	65	Yes

RCS: retrospective cohort study; PCS: prospective cohort study; RCT: randomized controlled trial; NA: not applicable; tPTX: total parathyroidectomy; tPTX + AT: total parathyroidectomy with autotransplantation; Other: subtotal parathyroidectomy and/or incomplete parathyroidectomy.

**Table 2.** Quality assessment of cohort studies.

Studies	Year	Selection	Comparability	Outcome	Score
Higgins et al.	1991	★★★★	⋆⋆	★⋆★	6
Nicholson et al.	1996	★★★★	★⋆	★★⋆	7
Coen et al.	2001	★★★★	⋆⋆	★★⋆	6
Ockert et al.	2002	★★★★	★★	★⋆★	8
Jofré et al.	2003	★★★★	⋆⋆	★★★	7
Shih et al.	2009	★★★★	★⋆	★★★	8
Conzo et al.	2012	★★★★	⋆⋆	★★★	7
Schneider et al.	2012	★★★★	⋆⋆	★★★	7
He et al.	2014	★★★★	★⋆	★★★	8
Liang et al.	2015	★★★★	★⋆	★★★	8

**Table 3.** Risk of bias of randomized control trial.

Study	Year	Random sequence generation	Allocation concealment	Blinding of participants and personnel	Incomplete outcome data	Selective reporting	Other bias
Schlosser et al.	2016	Low risk	High risk	Unclear	Low risk	Unclear	Unclear

There were eight retrospective cohort studies, two prospective cohort studies and one RCT. The follow**-**up period was from 4 months to 13 years. The sample size of these included studies ranged from 20 to 585. Three studies were conducted in Germany, three in China, two in UK, two in Italy, one in Argentina. Ten studies [12–21] described the indications for PTX. Only one study [22] described the etiology of sHPT. The NOS score of 10 studies was >5 stars, which were considered high quality for admission.

### Meta-analysis

#### Surgical complications

**Figure 2.**
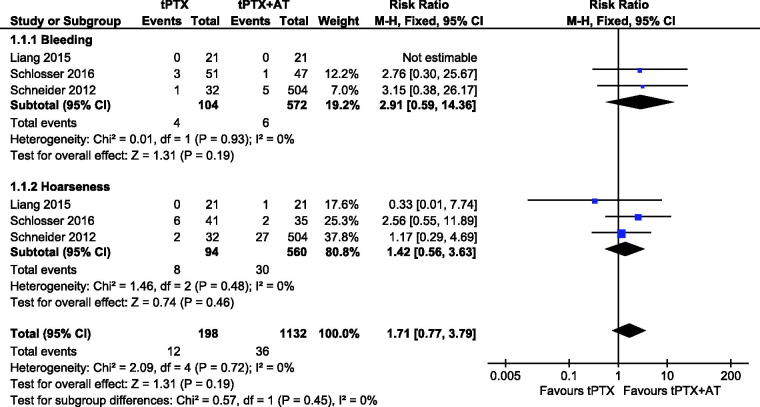
Forest plot for surgical complications.

Three studies [13,14,17] reported data for bleeding, 4/104 (3.8%) for the tPTX group and 6/572 (1.0%) for the tPTX + AT group. There was no statistically significant difference between tPTX and tPTX + AT (RR, 2.91; 95% CI, 0.59–14.36; *p* = .19).The heterogeneity between the studies was not substantial (*p* = .93, *I*^2^ = 0%), so we used a fixed-effects model for analysis. Three studies [13,14,17] described voice hoarseness, 8/94 (8.5%) for the tPTX group and 30/560 (5.4%) for the tPTX + AT. There was no significant difference between the two groups (RR,1.42; 95% CI, 0.56–3.63; *p* = .46). There was no substantial heterogeneity (*p* = .48, *I*^2^ = 0%), so we used a fixed-effects model for it. These surgical complications between studies had no statistical difference (RR, 1.71; 95% CI, 0.77–3.79; *p* = .19) (Figure 2).

#### All-cause mortality

**Figure 3.**
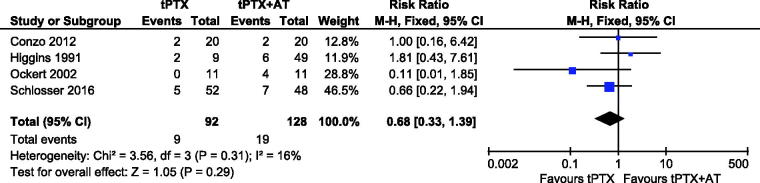
Forest plot for all-cause mortality.

Data regarding to all-cause mortality were reported in four studies [14,15,19,21], nine patients in tPTX group and 19 patients in tPTX + AT group. Prevalence of mortality in the tPTX group and tPTX + AT group was 9.78% (9/92) and 14.84% (19/128), respectively. Although the all**-**cause mortality in the tPTX + AT group was higher than that of the tPTX group, meta**-**analysis showed no significant difference (RR, 0.68; 95% CI, 0.33–1.39; *p* = .29). There was no substantial heterogeneity between the studies (*p* = .31, *I*^2^ = 16%), so a fixed-effects model was used for the meta-analysis (Figure 3).

#### Reoperation because of persistence or recurrence of sHPT

**Figure 4.**
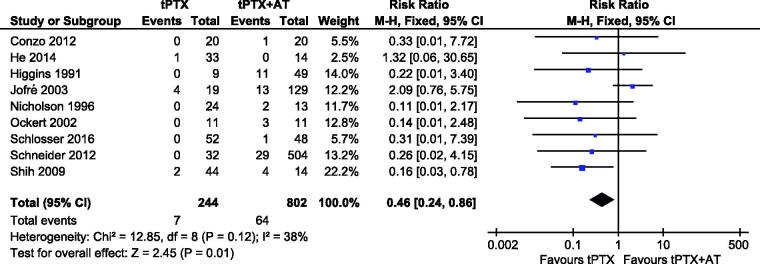
Forest plot for reoperation because of persistence or recurrence of sHPT.

There was no substantial heterogeneity among the studies (*p* = .12, *I*^2^ = 38%), so finally we accepted a fixed-effects model for the meta-analysis. Nine studies [12–16,18–21] reported data about reoperation, 244 patients in the tPTX group and 802 patients in the tPTX + AT group. The tPTX group had 7 events (2.87%), whereas another group had 64 events (8.0%). The tPTX + AT group had a higher prevalence of reoperation and simultaneously there was a significant difference (RR, 0.46; 95% CI, 0.24–0.86; *p* = .01) (Figure 4).

#### Persistence of sHPT

**Figure 5.**
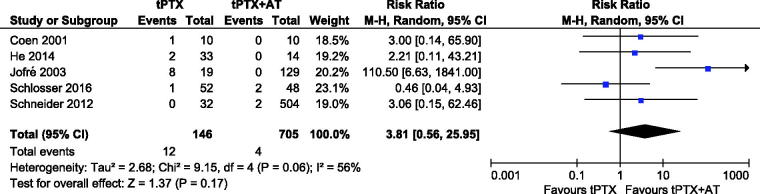
Forest plot for persistence of sHPT.

Data about persistence of the disease were reported in five articles [13,14,18,20,22]. Twelve events were noted in the tPTX group, whereas four events were observed in the tPTX + AT group. The meta**-**analysis showed no significant difference (RR, 3.81; 95% CI, 0.56–25.95; *p* = .17). The heterogeneity between the two groups was substantial (*p* = .06, *I*^2^ = 56%), so we accepted a random**-**effects model for the analysis (Figure 5).

#### sHPT recurrence

**Figure 6.**
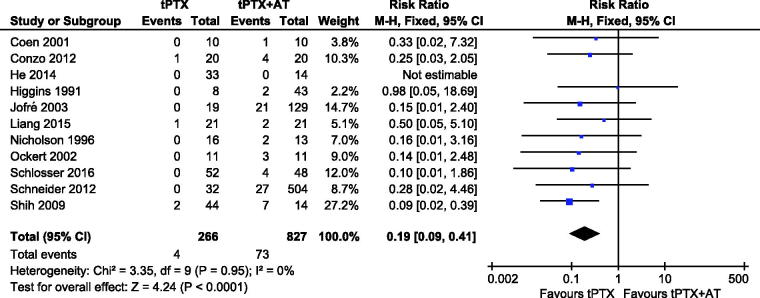
Forest plot for sHPT recurrence.

A meta-analysis on 11 studies [12–22] showed that patients in the tPTX group had a significantly lower risk of disease recurrence compared with the tPTX + AT group (RR, 0.19; 95% CI, 0.09–0.41; *p* < .0001) (Figure 6). There was no substantial heterogeneity among studies (*p* = .95, *I*^2^ = 0), so a fixed-effects model was used for the meta-analysis.

#### Hypoparathyroidism

**Figure 7.**
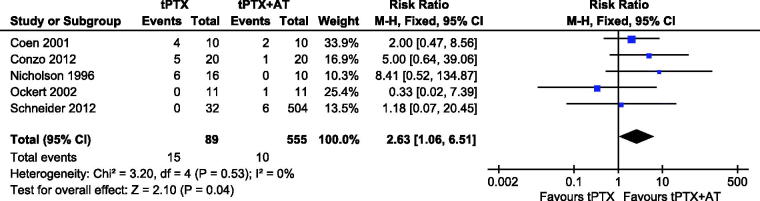
Forest plot for hypoparathyroidism.

Five studies [13,15,16,21,22] reported on hypoparathyroidism. There was no heterogeneity (*p* = .53, *I*^2^ = 0), so a fixed-effects model was used for this meta-analysis. Results showed no significant difference between studies (RR, 2.63; 95% CI, 1.06–6.51; *p* = .04) (Figure 7).

#### Symptomatic improvement

**Figure 8.**
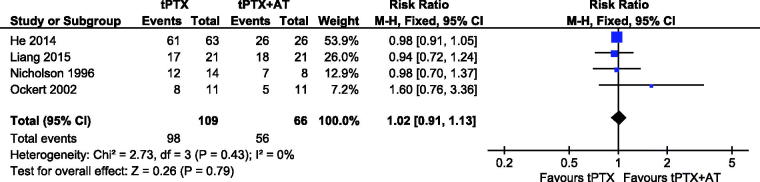
Forest plot for symptomatic improvement.

Four studies [15–17,20] reported on symptomatic improvement. A fixed-effects model was used for the analysis because heterogeneity was absent (*p* = .43, *I*^2^ = 0). Results showed no significant difference between tPTX and tPTX + AT (RR, 1.02; 95% CI, 0.91–1.13; *p* = .79) (Figure 8).

#### Publication bias

**Figure 9.**
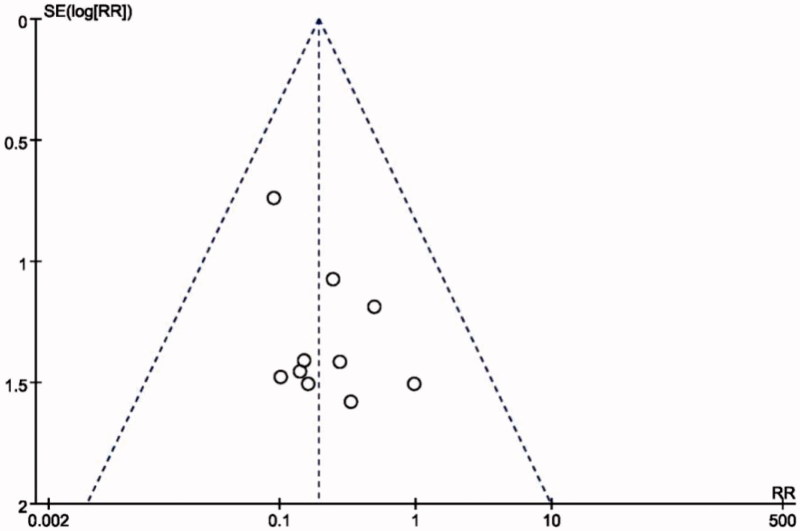
Funnel plot for sHPT recurrence.

A funnel plot was used to assess publication bias. There was no evidence of publication bias because the funnel plot of standard error by effect estimate of recurrence showed all studies lay within the limits (Figure 9).

#### Sensitivity analyses

A sensitivity analysis for disease persistence were used to determine the dependability of our results by accepting the leave-one-out approach. The heterogeneity of all five studies was significant (*p* = .06, *I*^2^ = 56%), and the heterogeneity disappeared (*p* = .70, *I*^2^ = 0) when the study by Jofré et al. [18] was excluded. The result continued to show no significant difference. When we removed each studies back, the pooled result indicating that no study dominated the analysis.

## Discussion

The relationship between sHPT and CKD has been well established [23]. sHPT is a common sequel of CKD that causes electrolyte disturbance and results in pathological fractures, cardiovascular disease and even death [24,25]. Despite recent advances in drug therapy, many patients still require surgery. The prevalence of surgery to treat sHPT with maintenance dialysis over 10–20 years is ≈15**–**38% [13]. PTX can improve the survival from sHPT as well as symptomatology and QoL [26,27].

Our systematic review was carried out by a comprehensive search for studies to appraise the safety and efficacy of two surgical approaches (tPTX and tPTX + AT) in sHPT. Finally, 11 studies involving 1108 patients with sHPT met the inclusion criteria and were enrolled in our meta**-**analysis. Our findings showed that both approaches were equally effective with regard to avoidance of surgical complications (bleeding and voice hoarseness), all-cause mortality, persistence of disease and symptomatic improvement. However, there were significantly fewer episodes of recurrence and reoperation because of recurrence/persistence of disease in the tPTX group compared with the tPTX + AT group. The tPTX group had an increased risk of hypoparathyroidism according to our meta-analysis.

The results of this meta-analysis showed no difference in the prevalence of surgical complications (bleeding and voice hoarseness) between the two groups. Both surgical procedures included removal of all parathyroid glands. After resection, tPTX + AT required another procedure: AT. The sites used for AT were the sternocleidomastoid muscle, brachioradialis muscle and tibialis anterior muscle [20,28,29]. Most surgeons chose the brachioradialis muscle for AT because this site is simple for reoperation. The AT site chosen would not increase the prevalence of bleeding and voice hoarseness.

The aim of every treatment is to improve survival or QoL. These two approaches had an identical effect on symptomatic improvement. Almost all patients suffered from bone pain and pruritus before surgery. Some studies indicated that these symptoms were relieved or disappeared the day after surgery regardless of the surgical approach. Also, the level of PTH in serum decreased dramatically after surgery. When the PTH level was >495 pg/mL, the mortality risk was increased by 25% [30]. PTX can reduce the prevalence of mortality in renal hyperparathyroidism [31]. However, the evidence from our study does not explain which one was better.

Persistent hyperparathyroidism is a common complication of surgery. There was no difference in persistence of hyperparathyroidism in our meta**-**analysis. The reasons for persistence can be ectopic and supernumerary parathyroid glands. Identification of ectopic parathyroid gland is challenging because parathyroid glands are not always in the same anatomic position. Gomes et al. [32] described the most common sites to be thyroid parenchyma (33.3%), thyroid**-**thymus conduit (18.5%) and thymus (14.8%).The prevalence of supernumerary parathyroid glands by random autopsies was ≈13% [33]. Akerstrom et al. [34] reported that 3% of patients had three parathyroid glands, 84% had four parathyroid glands and 13% had supernumerary glands. Many supernumerary glands were located in thymus tissue. The prevalence of persistent hyperparathyroidism could not be improved by surgical methods alone.

Recurrent hyperparathyroidism was a serious complication after PTX. Results showed that tPTX could reduce the risk of recurrence better than tPTX + AT. Recurrent hyperparathyroidism could not be avoided in patients undergoing maintenance dialysis with time. In 80% of patients after TPTX + AT, disease recurrence was in the graft, and the other 20% was in the neck [35]. Zhong et al. [36] reported that tPTX + AT is associated with sHPT recurrence. Melck et al. [37] suggested that tPTX + AT should be abandoned as sHPT treatment because of frequent recurrence. Tominaga et al. [38] stated that graft**-**dependent recurrence could be controlled by removing the autograft.

The pooled result of included studies suggested that tPTX could increase the risk of hypoparathyroidism. However, adynamic bone disease or hypocalcemia were absent. This procedure was conducted by removal of all parathyroid glands. However, PTH levels could be measured in many patients after tPTX because PTH may be secreted from the thymus gland [39]. Conzo et al. [40] suggested that hypoparathyroidism could be treated by pharmacological means.

### Strengths and limitations of our meta-analysis

The strengths of our meta**-**analysis were explicit inclusion criteria, broad search strategy and independent assessment of eligibility by two reviewers. This meta**-**analysis had three main limitations. First, most of the included studies had small sample**-**sized cohort studies, so the results of our study may not be adequate to judge the safety and efficacy of tPTX and tPTX + AT. Second, some studies reported recurrent and persistent hyperparathyroidism, but the definitions of recurrence and persistence were not mentioned in most of them. Third, some studies described long**-**term serum levels of calcium, phosphate, alkaline phosphatase and PTH, but the authors presented them in different ways (histogram, line graphs), so we could not draw conclusions on changes in biochemistry.

## Conclusions

Our meta**-**analysis suggests that tPTX and tPTX + AT are effective surgical approaches to the treatment of sHTP. They also show the apparent benefits of tPTX on sHPT recurrence and reoperation. Hence, tPTX could be the best way to treat uncontrolled sHPT. Nevertheless, tPTX increases the risk of hypoparathyroidism compared with tPTX + AT. Surgeons counseling patients should consider the benefits and disadvantages of tPTX versus tPTX + AT. To guide future work in this area, large multicenter RCTs comparing the two approaches are necessary.
